# Folic acid depletion as well as oversupplementation helps in the progression of hepatocarcinogenesis in HepG2 cells

**DOI:** 10.1038/s41598-022-21084-9

**Published:** 2022-10-05

**Authors:** Renuka Sharma, Taqveema Ali, Jyotdeep Kaur

**Affiliations:** Department of Biochemistry, Post Graduate Institute of Medical Education and Research, PGIMER, Chandigarh, India

**Keywords:** Biochemistry, Cancer, Molecular biology, Oncology

## Abstract

Folate ingestion below and above the physiologic dose has been shown to play a tumorigenic role in certain cancers. Also, excessive folate supplementation after establishment of pre-established lesions led to an advancement in the growth of a few tumors. However, such information has not yet been achieved in the case of HCC. In our study, HepG2 cells were administered with three different concentrations of folic acid i.e. folic acid normal (FN) (2.27 µM), folic acid deficient (FD) (no folic acid), folic acid oversupplementation (FO) (100 µM) for 10 days. Intracellular folate levels were assayed by Elecsys Folate III kit based method. The migratory and invasive abilities were estimated by transwell migration and matrigel invasion methods respectively. FACS was done to evaluate cell viability and apoptosis. Agarose-coated plates were used to access cancer stem cells (CSCs) number. Quantitative RT-PCR and western blotting approaches were used for gene and protein expression of certain tumor suppressor genes (TSGs), respectively. FD cells depicted increased migration, invasion, apoptosis, necrosis and decreased cell viability, CSCs. On the other hand, FO cells showed increased migration, invasion, cell viability and number of CSCs and decreased apoptosis and necrosis. TSGs revealed diminished expression with both FA modulations with respect to FN cells. Thus, FA deficiency as well as abundance enhanced the HCC progression by adapting different mechanisms.

## Introduction

Folate is a water soluble B9 vitamin, naturally occurring in green leafy vegetables and fruit juices. Folic acid (FA) is the oxidized form of this vitamin that is artificially added to fortified foods items^1^. Folate is usually considered safe and assumed to be entirely beneficial and a perfect food element helping in disease prevention. Based on its functional importance, any sort of imbalance in folate levels in the cells can lead to severe consequences. Folate deficiency is a highly prevalent deficiency all over the globe. It was the most frequent vitamin deficiency in the US, approximately affecting up to 60% of young or elderly people and10% of the general adult population belonging to low socio-economic groups^2^ before the food fortification program started in 1998. Folate deficiency in pregnant women has been linked with the occurrence of neural tube defects (NTDs) in neonates. It is also associated with megaloblastic anemia, cardiac problems^2^ atherosclerosis^3^, neuropsychiatric disorders^4,5^ and the risk of numerous cancers like colorectal, lungs, ovarian, prostate, etc. Poor dietary consumption, impairment in intestinal absorption e.g., in celiac disease, short bowel syndrome, amyloidosis and impaired renal tubular reabsorption, pregnancy, achlorhydria, hemolytic anemia, alcoholism, dialysis etc. can cause folate deficiency^1^. The facts from the post-fortification period have provided potential proof in decreasing NTD cases due to mandatory folic acid fortification ^6^. The efficacy of these programs in improving folate status proved quite striking, with a spectacular increase in blood folate concentrations in the USA and Canadian populations. Preliminary reports confirmed a reduction of about (∼15–50%) in the NTDs incidence in USA and Canada^7^. The circulatory folate concentrations illustrated inverse correlations with three aspects of tumor expansion: the size of the tumor^8^, its multiplicity and metastasis^9^.

In developing countries like India, FA is administered during the preconception period and through the gestation period at a much higher level than the recommended one according to WHO guidelines (600 μg vs. 5 mg)^10^. The consumption of FA from fortified food items (100–200 µg/d) collectively with the usage of supplements (which adds an extra 400 µg FA/standard multivitamin preparation) and ingestion of nutrition bars and drinks (which is frequently supplemented with 400 µg FA/serving) buildup a condition of folate oversupplementation in the population, indicating that folate oversupplementation conditions do prevail among humans knowingly or unknowingly^11^. In context to prostatic tumors, the Aspirin/Folate Polyp Prevention Trial has reported a raised risk of malignancies^12^ and higher circulatory folate post androgen deprivation found associated with poor prostatic tumor-specific survival^13^. Recent epidemiological data have depicted that FA supplementation might promote the progression of established breast malignancies in an animal model^14^. Also, a systematic review concluded that increased consumption of folate reduced mammary tumor risk for females consuming higher content of alcohol, but not for those having lower alcohol intake^15^. Studies conducted in animal models of colorectal tumors reported that FA administration prohibits tumor growth in normal tissues but encourages cancer progression if tumor lesion has been established^16–18^. Thus the time of folate intervention is significant in either providing an effectual and safe chemo-preventive role against carcinogenesis or as a promoter in favor of tumor development^17^.

In addition to the role of folate in nucleotide synthesis and methylation reactions, folate-dependent pathways play a major role in cell motility, including cancer cell migration as well as invasion^19^. A study depicted that colorectal cells when grown in inadequate FA exhibited impaired colonosphere formation^20^ and improved invasive properties of colon tumor cells mediated by activation of Shh signaling pathways via hypomethylation^21^. However, conflicting results reported in the cells of head and neck squamous cell carcinoma (HNSCC)^22^ and breast tumors^19^ which when depleted with methyl donors showed reduced growth, mobility and increased apoptosis^22^. Consistent with this, the dividing ability of embryonic stem cell was improved on treatment with folate^23^. Folate pathways are also known to promote invasiveness in prostate cancer cells^24^ and motility in A549 lung carcinoma cells affecting actin dynamics^25^ which might be due to the regulation of methylation of the genes involved^26^. In the case of liver, a study suggested that cells grown in folate-depleted media appeared to be more mobile and generated more number of spheres^27^. Apart from this, the effect of FA modulations in HCC cells has not been studied.

The molecular link between different doses of folate treatment and cancer hallmarks is also not well established. Till date, literature suggests that the effect of FA on the cancer cell is cell-type specific. So, in the present study we tried to decipher the effect of FA modulations on certain cancer hallmarks and expression of tumor suppressors in HepG2 cells, which might be help in identifying the underlying mechanism behind the HCC growth and its metastatic spread.

## Results

### Effect of FA deficiency and oversupplementation on cell morphology

**Figure 1:**
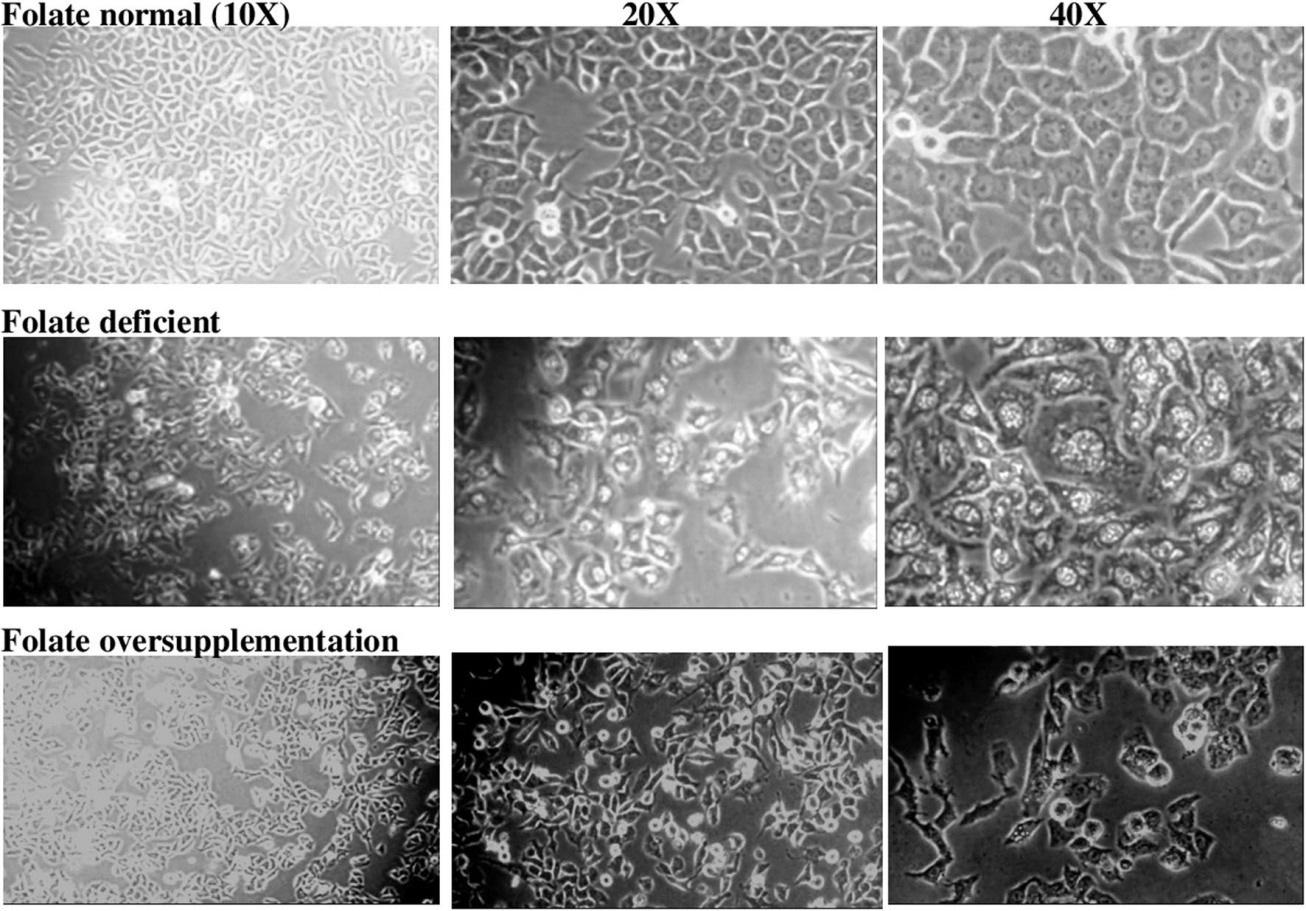
Effect of folic acid deficiency and oversupplementation on the morphology of HepG2 cells under the magnifications i.e. 10×, 20× and 40×. FN-Folic acid normal, FD-Folic acid deficient, FO-Folic acid oversupplemented.

HepG2 a liver cancer cell line possesses high proliferation rates with epithelial-like morphology. We observed that after treating these cells with different concentrations of FA, cells treated with the high FA were similar in morphology to FN cells. Cells in FA deficient media showed different morphology. The nucleus of FD cells seems to be enlarged, showing a disintegrated pattern or chromatin condensation as compared to the other two groups (Fig. 1).

### Effect of FA deficiency and oversupplementation on intracellular folate levels

**Figure 2:**
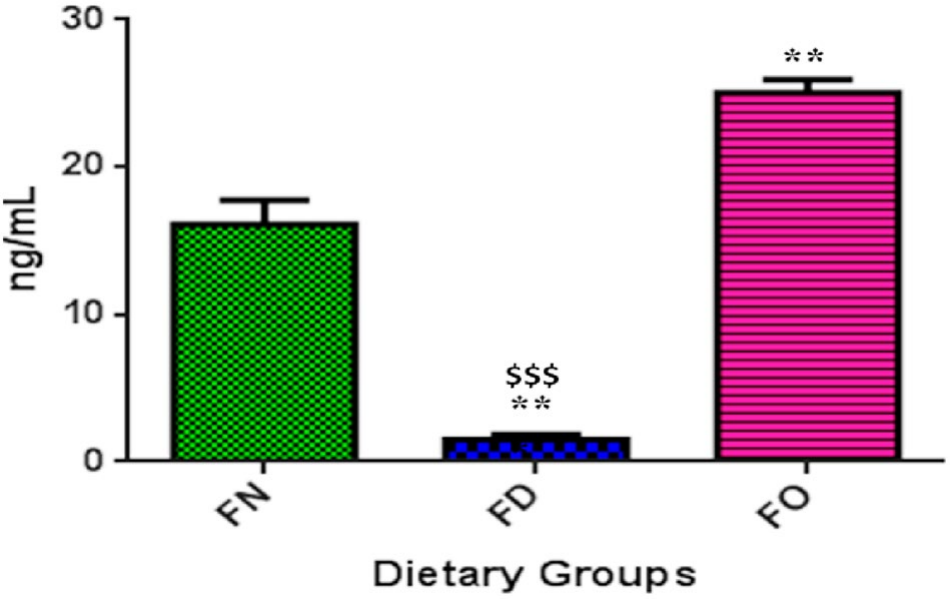
Effect of folic acid deficiency and oversupplementation on intracellular folate levels in HepG2 cells. **P < 0.01 versus FN, ^$$$^*P* < 0.001 versus FO; FN-Folic acid normal, FD-Folic acid deficient, FO-Folic acid oversupplemented.

In order to determine whether the treatment of FA deficiency and oversupplementation established the intracellular folate levels accordingly, we estimated folate levels in HepG2 cells. We observed that folate concentration in FD cells was significantly decreased (1.5 ± 0.6 ng/mL; *P* < 0.01) and in FO cells, it was significantly increased (25 ± 1.6 ng/mL; *P* < 0.01) when compared with FN cells (16.2 ± 2.9 ng/mL). In an intergroup comparison between FD and FO, markedly reduced (*P* < 0.001) folate levels in FD cells in comparison with FO cells were found (Fig. 2).

### Effect of FA deficiency and oversupplementation on cell viability and apoptosis

#### Cell viability

**Figure 3:**
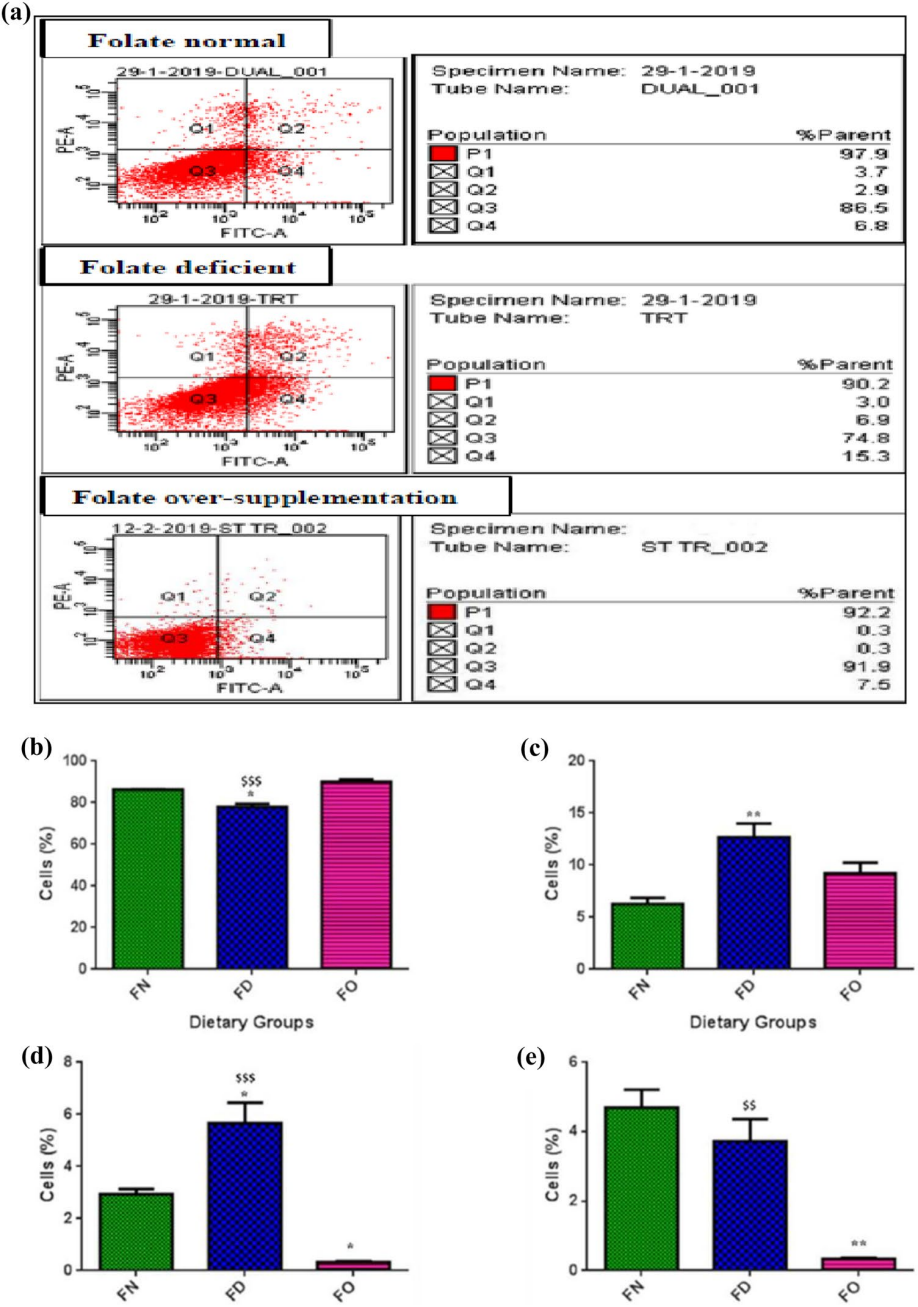
Effect of folic acid deficiency and oversupplementation in HepG2 cells on (**a**) PI-Annexin assay by flow cytometry. (**b**) Cell viability (**c**) Early apoptosis (**d**) Late apoptosis (**e**) Necrotic cells. *P < 0.05, **P < 0.01 versus FN, ^$$^*P* < 0.01, ^$$$^*P* < 0.001 versus FO; FN-Folic acid normal, FD-Folic acid deficient, FO-Folic acid oversupplemented.

The representative image of flow cytometric analysis was depicted in Fig. 3a. Viable cells were more in number in group treated with excess FA, although this increase was not statistically significant. A significantly less (*P* < 0.05) number of viable cells was seen in the FD group. The number of viable cells in the FD subset was observed to be reduced (*P* < 0.001) as compared to the FO group. Excess FA supplementation might have led to an increase in the number of proliferating cells (Fig. 3b).

#### Apoptosis

Upon FACS, FO cells revealed that the number of cells undergoing necrotic and late apoptotic changes were significantly low (*P* < 0.01; *P* < 0.05 respectively), as was the case with cells with early apoptotic changes albeit, non-significantly. Cells grown under conditions of FA scarcity demonstrated an increase in the number of cells with early (*P* < 0.01) plus late (*P* < 0.05) apoptotic alterations. A significant increase in the number of late apoptotic (*P* < 0.001) and necrotic cells (*P* < 0.01) in the FD treated group was observed in comparison to FO. FO treatment resulted in a decrease in the number of necrotic and late apoptotic cells. Whereas with FA scarcity, cells with early and late apoptotic changes were increased, indicating more apoptosis under deficiency treatment {Fig. 3c–e}. Annexin V-positive and PI-positive cells were considered as the late apoptotic cells.

### Effect of FA deficiency and oversupplementation on cell migration

**Figure 4:**
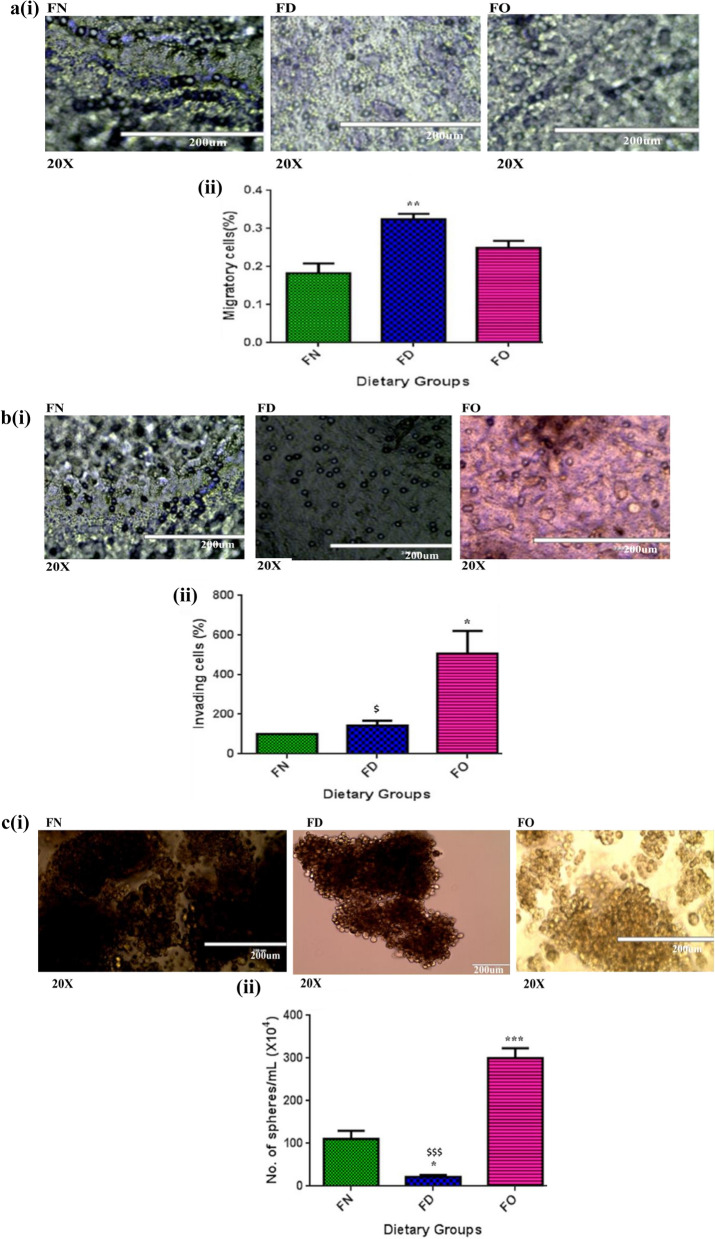
Effect of folic acid deficiency and oversupplementation in HepG2 cells. (**a**(**i**)) Representative images of migratory cells under microscope under 20× magnification (**a**(**ii**)) Quantification of migratory cells. (**b**(**i**)) Representative images of invasive cells under microscope under 20X magnification (**b**(ii)) Quantification of invading cells. (**c**(**i**)) Representative images of spheres under microscope under 20× magnification (**c**(**ii**)) Quantification of spheres formed. 20X = 200 µm.*P < 0.05, **P < 0.01, ***P < 0.001 versus FN, ^$^*P* < 0.05, ^$$$^*P* < 0.001 versus FO; FN-Folic acid normal, FD-Folic acid deficient, FO-Folic acid oversupplemented.

On staining, the migratory HepG2 cells with crystal violet, the cell number was found significantly more (*P* < 0.01) for FD cells when compared with FN cells. However the number of migratory cells also increased for FO cells but this change was not statistically significant. Thus, FA deficit conditions increased the migratory activities in HepG2 cells, thus favoring metastasis {Fig. 4a(i&ii)}.

### Effect of FA deficiency and oversupplementation on cell invasion

The matrigel invasion assay revealed that the number of invading cells in the FD group was almost similar to cells treated with normal FA concentrations. However, the invading cell count was found significantly raised (*P* < 0.05 each) for FO cells when compared with FN or FD, respectively. Therefore, treatment with excessive FA increased the invading properties of HepG2 cells {Fig. 4b(i&ii)}.

### Effect of FA deficiency and oversupplementation on sphere formation

Cancer stem cells have the property of proliferating and surviving even in such harsh conditions. One sphere results from proliferation of single stem cell^28^. We observed a significantly lesser (80.7%; *P* < 0.05) number of spheres in the cells grown without FA compared to FN cells in sphere formation assay. A marked elevation of 63% (*P* < 0.001) in sphere count was observed for cells administered with an additional quantity of FA when compared with FN cells. On the evaluation of FD cells with FO cells, a considerably 14-fold lesser number of spheres were seen in the FD fraction (*P* < 0.001) of HepG2 cells. FA deficiency decreased and oversupplementation increased number of spheres formed, indicating beneficial effect of excessive FA for cancer stem cell survival {Fig. 4c(i&ii)}.

### Effect of FA deficiency and oversupplementation on gene expression of TSGs

RNA quality was checked on 1.5% agarose gel. The intact bands of 28S and 18S rRNA species on the gel depicted a good quality of RNA. Primer sequences used in RT-PCR for all the studied gene has been represented in Table S1 in supplementary information.

**Figure 5:**
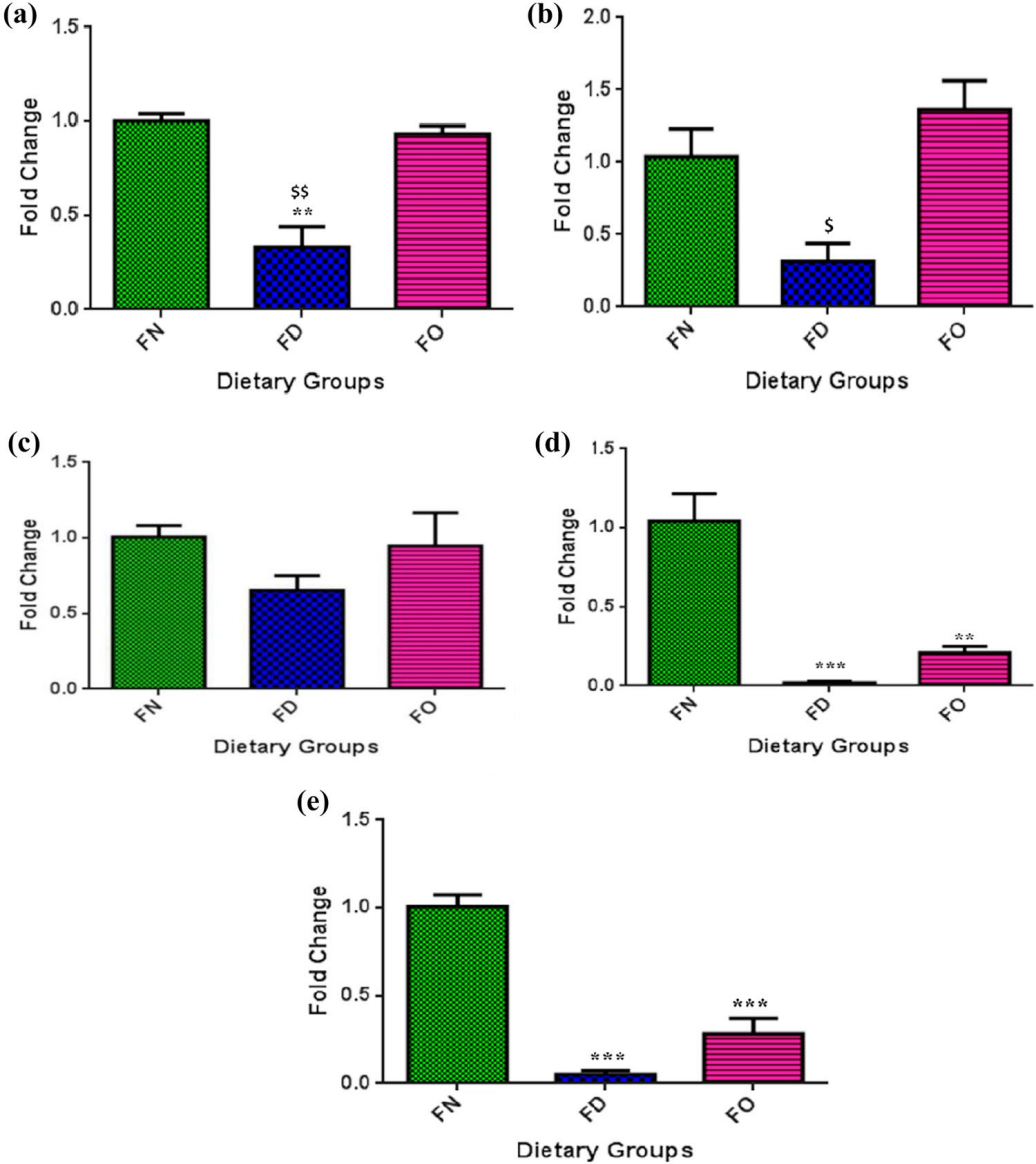
Effect of folic acid deficiency and oversupplementation on the gene expression of tumor suppressor genes: (**a**) *DPT* (**b**) *RUNX3* (**c**) *p16* (**d**) *RASSF1A* and (**e**) *SOCS1*. *18S* was used to normalize expression of studied genes. **P < 0.01, ***P < 0.001 versus FN, ^$^*P* < 0.05, ^$$^*P* < 0.01 versus FO; FN-Folic acid normal, FD-Folic acid deficient, FO-Folic acid oversupplemented.

In HepG2 cells, mRNA expression studies revealed a decrease in the expression of *DPT* gene in the cells which were allowed to grow under the conditions of no FA (FD) when compared with the cells which were grown under normal concentration of FA (FN). For FO cells, no noticeable change in the expression was observed (Fig. 5a). Relative to FO, cells of FD treatment illustrated a decline in *DPT* expression.

Similar to *DPT* gene, mRNA levels of *RUNX3* depicted a significant decrease in the expression in FD cells in comparison with FO cells. No significant effects were observed in FO cells when compared to FN cells (Fig. 5b). Gene expression of *p16* was found to be decreased, albeit non-significantly, in FD and unaltered in FO cells with respect to FN cells (Fig. 5c).

In contrast, *RASSF1A* mRNA levels revealed a significant decrease in both the treated cell groups i.e. in FD and in FO cells with respect to FN cells (Fig. 5d). Similarly, diminished gene expression in FD as well as FO groups was observed for *SOCS1* gene relative to cells administered with normal FA quantity (Fig. 5e). Fold change in gene expression of TSGs is detailed in Table S2 in supplementary information.

### Effect of FA deficiency and oversupplementation on protein expression of TSGs

**Figure 6:**
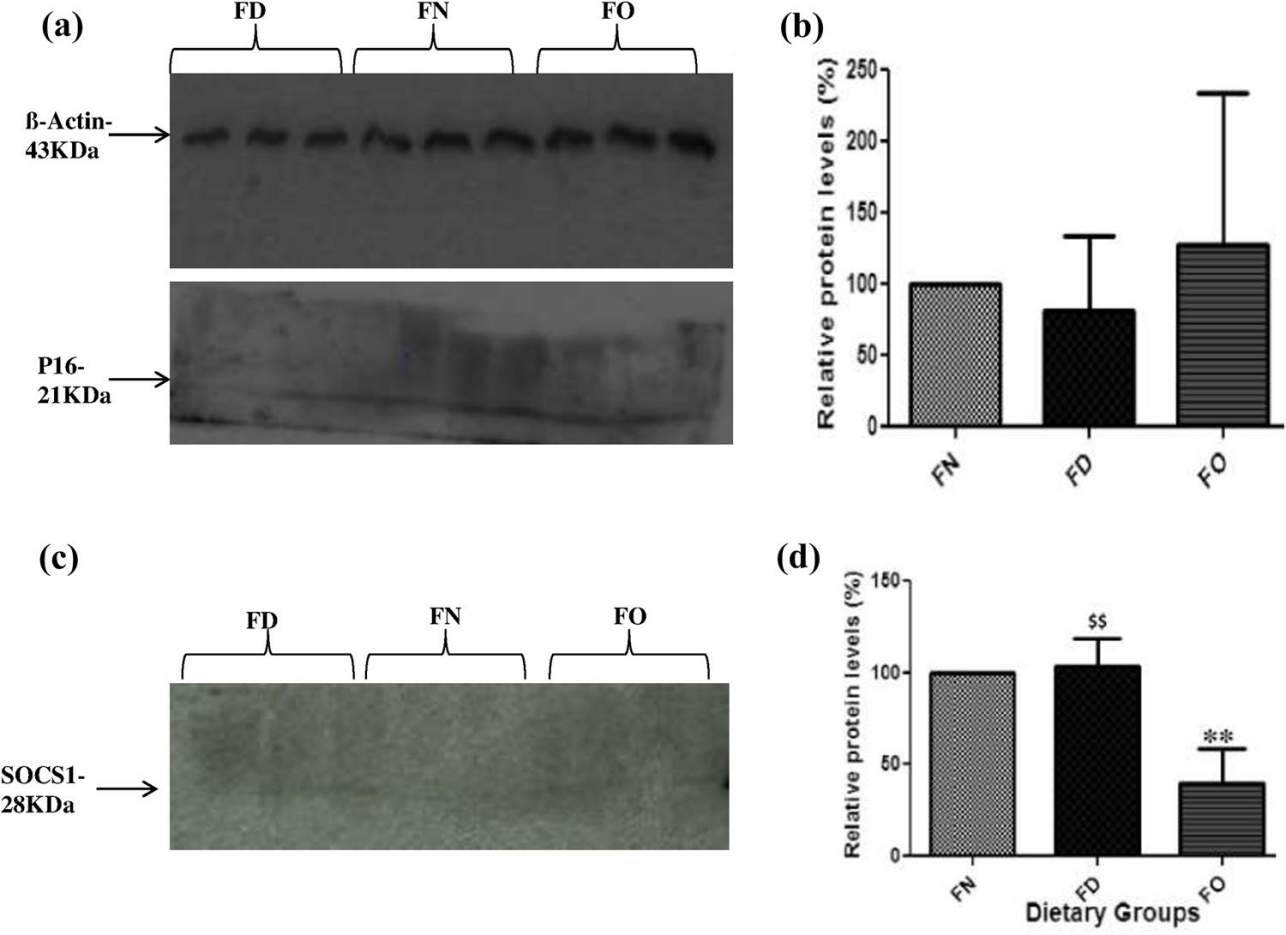
Effect of folic acid deficiency and oversupplementation on protein expression in HepG2 cells (**a**) Representative images of β-Actin & p16 blot (**b**) Densitometric analysis of the p16 blot. (**c**) Representative image of SOCS1 blot (**d**) Densitometric analysis of the SOCS1 blot. **P < 0.01 versus FN, ^$$^*P* < 0.01 versus FO; FN-Folic acid normal, FD-Folic acid deficient, FO-Folic acid oversupplemented.

Blot images were normalized with the expression of β-actin protein. The representative images of the β-actin, p16 and SOCS1 blots have been shown in (Fig. 6a,c). Similar to gene expression, densitometric analyses of protein levels of p16 depicted a decrease in FA deficit but the change was insignificant (Fig. 6b). SOCS1 protein levels were significantly decreased in FO cells when a comparison was made with FN cells. No considerable changes were observed in FD cells when the evaluation was done with FN cells. However in comparison with FO cells, cells of deficient fraction illustrated an increase in its protein expression (Fig. 6d). Numerical data for the same has been depicted in Table S3 in supplementary.

### Data retrieval of TSGs from TCGA database

**Figure 7:**
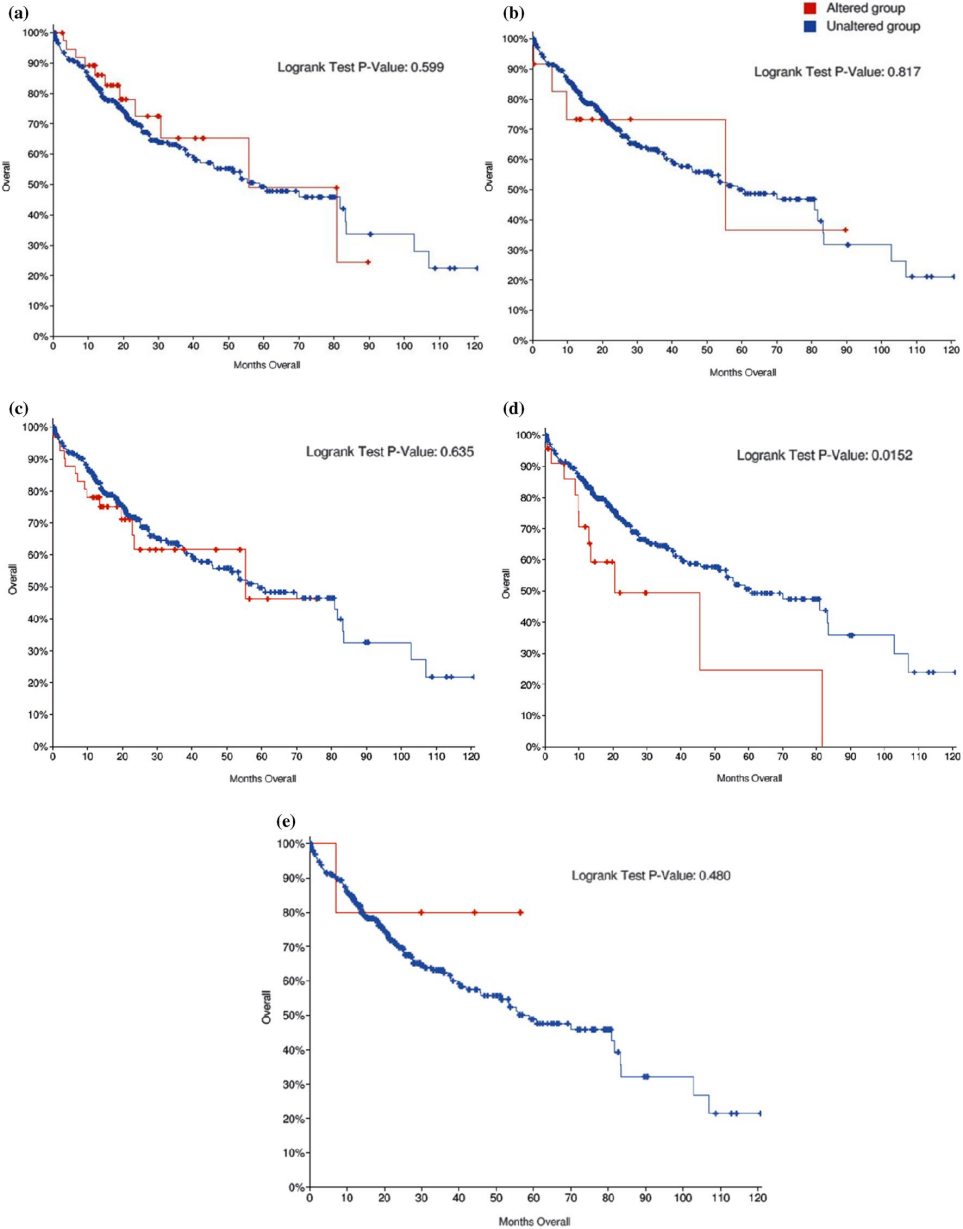
Survival curves retrieved from TCGA database for all the studied genes; (**a**) *DPT* (**b**) *RUNX3* (**c**) *p16* (**d**) *RASSF1A* and (**e**) *SOCS1*.

The survival curves from TCGA database depicted a decrease in patient survival months having an alteration in any of these genes, viz. *DPT, RUNX3, P16, RASSF1, SOCS1* (Fig. 7a-e). However, the survival was significantly reduced (*P* < 0.015) with alterations in *RASSF1A* gene.

## Discussion

DNA synthesis and methylation are the vital cellular processes that claim the presence of folate^29^. Dysregulation in folate levels has been linked with induction of base substitution, gene deletions and DNA strand breaks as well as altered gene expression^30^. Folate probably plays a double role in tumorogenesis, initially as a protector of DNA damage before the onset of neoplastic transformation and later behave as a promoter for cancer growth by increasing proliferation and invasion-like processes^16,31^. We attempted to study how modulation of folate levels in HCC cells affects the various hallmarks of cancer.

As expected, FA deficiency and it’s over-administration resulted in a significant decrease and increase in folate levels, respectively, in HepG2 cells. The previous studies also showed a similar reduction in folate levels under FD treatment^32–35^. Importantly, low folate levels have been linked with the risk for certain tumors^36^.

HepG2 cells, when grown without FA for ten days, showed enlarged nuclei relative to the other two groups, which might be due to nuclear chromatin condensation. These observations were in agreement with the previous study on the same cell type^37^. Moreover, a lack of correlation between nuclear dimension and ploidy has been depicted for carcinomas of the urinary bladder, colorectum, mammary glands, lung, skin, cervix, and prostate^38^. Morphologically FN and FO cells were indistinguishable from each other. However it was earlier seen that FO exerted a protective effect on hexagonal parenchymatous liver cells, thus improving liver morphology in aged rats^39^.

For HepG2 cells, we achieved a reduced number of live cells in FD when stained with PI-annexin dye. This decreased viability was reported earlier too in the same cell type^37^. Also experiments with FD-conditioned media with colon cells illustrated the same results^40^. Hepatoma cells that were supplemented with excess FA presented with increased counts of viable cells in our study. A previous finding also suggested that FO markedly enhanced the polyp number and tumour burden in the intestine^41^. In a contrasting observation, FA treatment (20 & 50 µM) inhibited nasopharyngeal cancer cell proliferation, suggesting that FA affects cell proliferation in a dose and cell type-specific manner^42^.

Our experiments with hepatic cells showed an increased migration under FD conditions, while extra FA did not significantly affect it. While studying invasion, FO cells showed a pronounced increase and decline in the invasion relative to FN and FD cells, respectively^27^. According to the previous findings, FD-conditioned SK-Hep1 and Mahlavu (the liver cancer cells) displayed a higher migration and invasion when compared with their respective control^25^. Contrasting evidences suggested that folate withdrawal inhibited migration and invasion of A549 cells^43^, while did not affect the migratory activity of A375 human melanoma cells. Also, Hearnden^22^ recently demonstrated that HNSCC cells in the absence of methyl donors showed extensively diminished cellular proliferation, impairment in cellular migration and increased apoptotic activities, indicating a less aggressive phenotype of the tumor. In line with our observation, the literature data suggested that FO can boost the growth and invasive potential of prostate carcinoma cells^44^ and modify the expression of genes related to cell adhesion, migration and invasion^45^. Folate treatment above physiological levels supports epithelial to mesenchymal transition in experimental mouse tumor cells and silencing of RhoA and Rac1, which serve as immediate sensors of folate intracellularly, inhibited the effects of folate on cellular migratory and invasive abilities^46^. These observations signify the cell and dose type-dependent effects of folic acid modulations.

Spheres in HepG2 were considerably reduced upon folic acid restriction and markedly increased upon its oversupplementation. A previous study on colon cells grown without adequate FA exhibited impairment in colonosphere forming ability^20^, similar to our results. In contrast to our findings, Sk-Hep1 and Mahlavu, under FD-conditioned media, generated a higher number of spheres, again stressing the fact that effects of folic acid deficiency are not only tissue-specific but also cancer type-specific, too^27^. A prior study reported the same findings that FA administration activated neural stem cell (NSC) proliferation in a dose-dependent manner and the plausible mechanisms were deciphered to be notch signaling pathway^23^. Thus, folic acid depletion can increase or decrease the number of tumorospheres generated, whereas folic acid oversupplementation raises the oncospheres, inferring the cell or tissue-specific role of folate modulations.

In HepG2 cells, TSGs transcript (*RASSF1A* and *SOCS1*) were found to be significantly decreased in both the dietary folate modulations relative to cells administered with normal amounts of FA. Similar to our study, dermatopontin (DPT), an extracellular matrix component^47^, was appreciably decreased in 202 HCC patients and can be correlated with metastasis and prognosis. Also, it inhibited cell motility in HCC mainly via α3β1 integrin, including diminished RhoA activity^48^. So, it could be possible that different folic acid modulations affect RhoA activity and lead to increased migration and invasion in our experiments. A study by Xirong et al., confirmed high expression of DPT in the fully developed liver and fetal life, while it decreased in HCC tissues^49^. Reduced *DPT* mRNA in our experiments was in concordance with findings in ApoE null mice, in which feeding mice with a combination of folate deficiency and a fat rich diet decreased DPT expression, which regulate differentiation, adhesion and migration abilities of the cell^50^. It is also among the top up-regulated genes in severe NAFLD^51^*,* NASH and fibrosis^52^. DPT expression was closely found associated with overall survival (OS) as well as with disease-free survival in HCC^48^, similar to the data we retracted from the database in September, 2020.

Also, the diminished expression of *RUNX3* correlates well with the ample literature- based evidence, which states that RUNX3 is lowered in HCC tissues and cell lines^53–55^. Restoration of *RUNX3* expression helps in lowering the number of stem cells by suppressing Notch signaling^56^ and controls metastatic spread via interacting with miR-186/E-cadherin/EMT in HCC^57^. Seventy percent of human HCCs revealed *RUNX3* hypermethylation^58^. In comparison with RUNX3-positive HCC, RUNX3-negative malignancies showed poor OS and relapse related survival^57^. A study reported raised *RUNX3* was reduced after folate repletion in keratinocytes^59^.

In FD-conditioned HepG2 cells, p16 expression was decreased at gene as well as protein levels relative to FN. Also, under folate oversupplemented conditions, p16 revealed non-significant changes in expression. The reduced p16 expression in FD cells was in concordance with a report which observed diminished *p16* mRNA due to hypermethylation in liver malignancies^60^. On exposure to folate/methyl deficiency, hypermethylation was seen for *p16* in hepatic tumors^61^. In HCC patients, high circulatory folate is associated with *p16* hypomethylation in the elderly Chilean population^62^. Insufficient folate consumption is associated with *p16* hypermethylation in an older population in colon^63^, head and neck^64^ and gastric tumors^65^. Folate withdrawal unaltered the methylation of *p16* in HCT116 and Caco-2 cells^66^.

In HepG2 cultured cells, the *RASSF1A* transcript was diminished for both FD and FO. Previous data confirmed *RASSF1A* and *p16* inactivation by aberrant methylation, a commonly occurring event in HCC^67^, while no change in *RASSF1A* methylation was identified for Hep3B and HepG2 cells^68^. The TCGA database depicted decreased survival in liver cancer patients with altered *RASSF1.* However, literature also evidenced the reverse findings that *RASSF1A* hypermethylation results in longer OS in HCC patients^69^. Another study depicted that the low folate diet in combination with increased alcohol intake was associated with hypermethylated *RASSF1A* in CRC subjects^70^. In a contrasting observation in lung cancer patients, blood cell folate was found associated with raised *RASSF1A* methylation^71^, whereas folate levels less actively influenced RASSF1A in breast cancer tissues^72^.

SOCS1, the negative regulator in cytokine signaling, showed expression in both treated groups in HepG2 cells in the present study. Prior studies have evidenced the same results that frequent CpG promoter methylation and its subsequent loss has been identified in HCC for SOCS1^73^. Previously, an immunohistochemical study illustrated intense homogeneous/ heterogeneous staining in the non-cancerous hepatic tissue as compared to HCC tissue which presented a noteworthy decrease with heterogeneous staining^74^. Also, in the rodent model of HCC induced by methyl deficient diet, epigenetic silencing of the *SOCS1* was observed^75^. Likewise, altered methylation and expression of SOCS1 was confirmed in HCC cell lines and upon restoration of the expression, suppressed expansion and anchorage-free cell growth were seen^76^. In line with this, a study referred to aberrant *SOCS1* methylation as the main event in the transformation of cirrhotic nodules into HCC^77,78^. Also, high folate intake was associated with raised SOCS1 methylation in CRC in European American individuals^79^. Thus, both folate modulations reduced the expression of the studied TSGs, which might lead to enhanced cancer progression. The aberrant expression of these genes might be due to altered promoter methylation. However, the limitation of our study is that the promoter methylation of the studied tumor suppressor genes was not determined in response to the modulation of folate levels.

Therefore, in HepG2 cells, FA deprivation led to decreased cell proliferation, number of CSCs, expression of TSGs and increased apoptosis, migration and invasion. On the other hand, excessive FA exposure to these cells led to increased cell proliferation, number of CSCs, migration, invasion and decreased apoptosis, necrosis and generally reduced expression of TSGs. All the said hallmarks together might result in increased cancer expansion under FO treatment. Hence FA modulations do affect the development of HCC differentially; FA deficient tumors might be less aggressive while FA oversupplemented ones be more hostile tumor types by employing differential regulation of TSGs and manipulating the pathways leading to altered cell proliferation, CSCs, apoptosis, migration and invasion.

## Materials and methods

### Cell culture

The human liver cancer cell line HepG2 was obtained from NCCS, Pune. HepG2 cells were cultured at 37ºC in an atmosphere of 5% CO_2_ and 95% relative humidity in 75cm^2^ cell culture flasks containing minimum essential medium (MEM) supplemented with 10% fetal bovine serum (FBS), sodium bicarbonate, penicillin (100,000U/L) and streptomycin (10 mg/L). These cells were obtained at passage 30 and used experimentally between passages 35–50. They were routinely passaged at 2-3 days interval. The medium was refreshed after 2 and 3 days of treatment for FN, FO and FD cells, respectively. Cells were regularly watched in order to see for their deadherance from the flask surface. After that, cells were seeded in 75cm^2^ cell culture flasks at a density of 200,000 cells per flask. Based on FA content administered to the cells, these were divided into three groups i.e. FN, FD and FO. FN or control cells were maintained under the conditions mentioned above in MEM media containing 2.27 μM folate concentration with 10% FBS. FD cells were grown on a commercially available folate-deficient medium supplemented with 20% dialyzed FBS. Cells were routinely monitored for any morphological changes. FO cells were grown on MEM media with FA concentration of 100 μM and 10% FBS^80^. All the treatments were carried out for five generations which is equivalent to 10 days of treatment.

### Estimation of intracellular folate levels in HepG2 cells by using Elecsys Folate III kit

For folate determination in cultured cells, the viability of cells was determined by trypan blue exclusion assay after harvesting. Thereafter, 10^4^ cells were lysed by heating at 100ºC for 5 min in folate extraction buffer. Samples were cooled and clarified by centrifugation and supernatant was incubated at 37ºC following the adding 0.25volumes of rat plasma conjugase for three hours in folate extraction buffer. After incubation, samples were boiled to precipitate protein and the solution was cooled and clarified by centrifugation. Further, folate levels were measured by using elecsys folate III kit on Elecsys and Cobas e analyzer. The detailed composition of the buffers used for folate estimation has been given in supplementary information.

### Cell viability and apoptosis by FACS

Annexin V/PI assay was used for measure the number of viable cells and apoptosis of HepG2 cells using FITC Annexin V Apoptosis Detection Kit II (BD, Pharmigen). After ten days of treatment, HepG2 cells were harvested by using trypsin–EDTA. Cells were suspended in 1X binding buffer at a concentration of 1 × 10^6^cells/mL in 100μL and were transferred into a FACS tube. Two microlitres (µL) of Annexin V-FITC were added to each tube and mixed well. The samples were incubated for 15 min at room temperature in the dark. Additional 400μL of 1X binding buffer was added to each tube and 5μL of PI was freshly added into the cell suspension just before analysis and mixed. The cells were analyzed on a fluorescence-activated cell sorter and data was analysed using Cell Quest software.

### Transwell migration assay

Cell migration assay was performed using 24well transwell chambers (8.0 μm pore size polycarbonate membrane 65 mm, 0.33cm^2^). After ten days of respective media and FBS treatment, 1X10^5^ HepG2 cells were seeded in the upper of transwell chamber with respective media. MEM supplemented with their respective FBS was added to the lower chamber as a chemoattractant. Following incubation for 24 h at 37 ºC, non-invading cells remaining on the upper surface were removed using a wet cotton swab, whereas cells on the lower surface were fixed with 0.1% crystal violet and 20% methanol-containing solution for 30 min and washed with 1X PBS twice and kept for drying for 20 min and then placed on a glass slide. Then cells migrating across the membrane were imaged in random visual fields under an inverted microscope. After that, crystal violet dye retained on the filters was extracted using 600 μL of 30% acetic acid and then OD was taken at 590 nm. The values obtained and the percentage of invasion and migration were calculated using the following formula: (OD of extracted crystal violet from treated cells migrating through inserts minus blank)/OD of extracted crystal violet from negative control minus blank) * 100. All the experiments were repeated at least twice.

### Matrigel invasion assay

For invasion assay, HepG2 cells were plated onto the top of a 30 μg/cm^2^ matrigel coated transwell chamber of 24-well plate in 100μL serum-free medium. The bottom well of the chamber contained 600 μL of MEM supplemented with its respective FBS. After incubating for 24 h, chambers were disassembled and stained, following the protocol as discussed above for migration assay.

### Sphere formation assay

To check the ability of cancer stem cells to proliferate, a sphere formation assay was done on low attachment plates. For low attachment, 6 well plates were coated with 1% agarose, allowed to solidify and kept under UV for sterilization for at least six hours. Then HepG2 cells were seeded at a density of about 3000 cells per well with their specific MEM and FBS. Cells were further incubated at 37 ºC for about 15 days. Meanwhile after 3–4 days of treatment, additional media and FBS were added along the sides of the wells without disturbing spheres. During incubation sphere formation was observed under a microscope and images were captured. After 15 days of incubation, spheres were counted under a microscope and images were captured for each group.

### Quantitative RT-PCR

From HepG2 cells, total RNA was isolated using TRIzol reagent (Ambion). Further reverse transcription was carried out by using Fermentas cDNA kit. RT-PCR was performed with gene-specific primers for tumor suppressor genes (TSGs), viz. *DPT, RUNX3, p16, RASSF1A* and *SOCS1* on Applied Biosystems StepOnePlusTM Real-Time PCR. *18 s rRNA* was used as endogenous control. Relative quantification of gene expression was done by using the comparative CT method (ΔΔCT).

### Western blotting

HepG2 cells were lysed using RIPA buffer. After quantification with bicinchoninic acid assay, equivalent quantities of protein were run on SDS-PAGE and blotted using the PVDF membrane. Polyclonal primary antibodies for p16 (catalogue no. PAA794Ra01) & SOCS1 (catalogue no. PAH158Ra01) were purchased from Cloud Clone Corporation USA. The primary antibodies against RASSF1A (sc-58470) & β-actin (sc-47778) were obtained from Santa Cruz, CA, USA. An enhanced chemiluminescence detection system was used for developing. Densitometric analysis of blots was done with Image J software.

### Data retrieval from TCGA database

For all the studied genes, we also tried to retrieve survival data from TCGA database in HCC patients available in this database till September 2020.

### Statistical analysis

For statistical analysis, of more than two groups, we performed one-way analyses of variance ANOVA (α = 0.05) followed by the Tukey post-hoc test. GraphPad Prism (v.6.0.1) was used for statistical analysis. Data were expressed as mean ± SEM and were considered statistically significant at *P* < 0.05.

## Supplementary Information

Supplementary Information.

## Abbreviations

FA: Folic acid
FD: Folic acid deficiency
FN: Folic acid normal
FO: Folic acid oversupplementation
HCC: Hepatocellular carcinoma
