# Supplementary material for: Folic acid depletion as well as oversupplementation helps in the progression of hepatocarcinogenesis in HepG2 cells
Source: Sci Rep. 2022 Oct 5;12:16617. doi: 10.1038/s41598-022-21084-9 (PMC9534894; doi:10.1038/s41598-022-21084-9)
Supplement: Supplementary file 1 — Supplementary Information. [file 41598_2022_21084_MOESM1_ESM.docx]

**Folic acid depletion as well as oversupplementation helps in the progression of hepatocarcinogenesis in HepG2 cells**

**Renuka Sharma, Taqveema Ali, Jyotdeep Kaur^*^**

Dept. of Biochemistry, PGIMER, Chandigarh

***Author for correspondence:**

**Dr. Jyotdeep Kaur**

**Department of Biochemistry,**

**Post Graduate Institute of Medical Education and Research, India.**

**Email Id:** [**jyotdeep2001@yahoo.co.in**](mailto:jyotdeep2001@yahoo.co.in)

**Telephone: +91-172-2755181**

**Fax: +91-172-2744401**

**Reagents used for folate estimation:**

1.Folate extraction buffer: 0.1M potassium phosphate buffer pH 7.0, 50mM ascorbic acid, 10mM 2-mercaptoethanol, 150mM NaCl, 1mM EDTA, 1mM EGTA, 1% Triton X100

2. Ascorbate-Phosphate buffer: Ascorbate 1mg/mL, 0.1M potassium phosphate buffer, pH 6.3 with NaOH

*Rat plasma as source of conjugase enzyme.

**Supplementary Table-S1: Primer sequences along with their annealing temperature used in mRNA expression study**

| **Gene** | **Forward primer 5'-3'** | **Reverse primer 5'-3'** | **AnnealingTemp. (^o^C)** |
| --- | --- | --- | --- |
| *RUNX3* | ACTGTGATGGCAGGCAATGA | CTGGCCACCTGGTTCTTCA | 60ºC |
| *DPT* | AGAGCCGCTACTTCGAGTCAGT | GGAATATGGGCACCTCTTGCT | 60ºC |
| *SOCS1* | TGGAGCCAGGACCTGAACTC | CCCCCAACCCCTGGTTT | 60ºC |
| *p16* | CATAGATGCCGCGGAAGGT | TCTAAGTTTCCCGAGGTTTCTCA | 58ºC |
| *RASSF1A* | AAATCTTGGAGACCCTGCAAAC | ATCCAAAACCTTTCCTCATAGATGA | 62ºC |
| *18S* | CGGCTACCACATCCAAGGAA | GGGCCTCGAAAGAGTCCTGT | 63ºC |

**Supplementary Table-S2: Gene expression (Fold change) of tumor suppressor genes by QRT-PCR**

| TSGs | FD vs FN(Fold change; significance) | FO vs FN(Fold change; significance) | FD vs FO(Fold change; significance) |
| --- | --- | --- | --- |
| DPT | 3.0 fold; p<0.01 |  | 2.8 fold; p<0.01 |
| *RUNX3* | - |  | 4.3 fold; p<0.05 |
| *RASSF1A* | 51 fold; p<0.001 | 4.8 fold; p<0.01 |  |
| *SOCS1* | 19.5 fold; p<0.001 | 3.5 fold; p<0.001 |  |

**Supplementary Table-S3: Protein expression (Fold change) of tumor suppressor genes by Western blotting**

| **Protein** | FO vs FN **(Fold change; significance)** | FD vs FO **(Fold change; significance)** |
| --- | --- | --- |
| SOCS1 | 2.5 fold;p<0.01 | 2.6 fold; p<0.01 |

Western blot images for beta actin, p16 & SOCS1 has been illustated in below.

**Beta-actin blot images**

**p16** **blot images**

**SOCS1** **blot images**
